# Gαi1/3 mediation of Akt-mTOR activation is important for RSPO3-induced angiogenesis

**DOI:** 10.1093/procel/pwac035

**Published:** 2022-08-12

**Authors:** Gang Xu, Li-na Qi, Mei-qing Zhang, Xue-yun Li, Jin-long Chai, Zhi-qing Zhang, Xia Chen, Qian Wang, Ke-ran Li, Cong Cao

**Affiliations:** Clinical Research Center of Neurological Disease, The Second Affiliated Hospital of Soochow University, Suzhou 215004, China; Department of Orthopedics, The Affiliated Lianyungang Hospital of Xuzhou Medical University, The First Affiliated Hospital of Kangda College of Nanjing Medical University, Lianyungang 222002, China; Clinical Research Center of Neurological Disease, The Second Affiliated Hospital of Soochow University, Suzhou 215004, China; Clinical Research Center of Neurological Disease, The Second Affiliated Hospital of Soochow University, Suzhou 215004, China; Clinical Research Center of Neurological Disease, The Second Affiliated Hospital of Soochow University, Suzhou 215004, China; Clinical Research Center of Neurological Disease, The Second Affiliated Hospital of Soochow University, Suzhou 215004, China; Clinical Research Center of Neurological Disease, The Second Affiliated Hospital of Soochow University, Suzhou 215004, China; Department of Anesthesiology, Children’s Hospital of Soochow University, Suzhou 215025, China; Department of Anesthesiology, Children’s Hospital of Soochow University, Suzhou 215025, China; The Affiliated Eye Hospital, Nanjing Medical University, Nanjing 210029, China; Clinical Research Center of Neurological Disease, The Second Affiliated Hospital of Soochow University, Suzhou 215004, China; The Affiliated Eye Hospital, Nanjing Medical University, Nanjing 210029, China

**Dear Editor,**

R-spondin3 (RSPO3) is essential for vascular development and angiogenesis. Analyzing RSPO3-knockout embryos revealed severe vascular defects in the placenta (Aoki et al. 2007). In both Xenopus and murine embryos, RSPO3 KO led to significant vascular defects (Kazanskaya et al. 2008) and embryonic death (Kazanskaya et al. 2008). In the placenta, RSPO3 could promote vascular endothelial growth factor (VEGF) expression (Kazanskaya et al. 2008). RSPO3 is a ligand of low-density lipoprotein receptor-related protein 6 (LRP6) and leucine-rich repeat G protein-coupled receptor 4 (LGR4) to form a multiple ligands-receptors-cluster with Wnt and frizzled (FZD), thereby activating and amplifying downstream β-catenin signaling (Jin and Yoon 2012; Tocci et al. 2020). RSPO3 neutralizes two *trans*-membrane E3 ubiquitin ligases, zinc and ring finger 3 (ZNRF3)/ring finger protein 43 (RNF43). The two could decrease cell-surface Wnt receptors (Jin and Yoon 2012; Tocci et al. 2020).

Besides Wnt/β-catenin signaling, Akt-mammalian target of rapamycin (mTOR) activation is also essential for angiogenesis by promoting endothelial cell growth, survival, metabolism, and protein synthesis, nitric oxide synthesis, migration and tube formation. Gu et al. (2020) have reported that RSPO3 can promote epithelial-mesenchymal transition in ovarian cancer via activating Akt cascade, which appeared to be independent of Wnt/β-catenin signaling. RSPO3 activated Akt signaling and promoted choriocarcinoma cell growth (Chen et al. 2020). However, whether Akt activation is important for RSPO3-induced angiogenesis and the underlying signaling mechanisms are largely unknown.

G protein inhibitory α subunits (Gαi proteins) binding to G protein-coupled receptors (GPCRs) inhibits adenylate cyclase and decreases cyclic AMP contents. Our group has previously discovered that Gαi1 and Gαi3 are pivotal signaling proteins required for Akt-mTOR activation by multiple receptor tyrosine kinases (Cao et al. 2009; Zhang et al. 2015; Liu et al. 2018; Marshall et al. 2018; Sun et al. 2018; Wang et al. 2021; Yao et al. 2022).

To test the potential functions of Gαi1 and Gαi3 in RSPO3-induced Akt-mTOR activation, we utilized wild-type (WT) and Gαi1 and Gαi3 double knockout (“Gαi1/3 DKO”) mouse embryonic fibroblasts (MEFs, see our previous studies Cao et al. [2009], Zhang et al. [2015] Marshall et al. [2018], Sun et al. [2018], Bai et al. [2021], and Wang et al. [2021]). MEFs were first treated with RSPO3 at gradually increased concentrations (20, 50 and 80 ng/mL) and cultured for 15 min. In WT MEFs, RSPO3 robustly increased phosphorylation of Akt (Ser-473), p70S6K1 (“S6K”, Thr-389) and S6 (Ser-235/236) (Fig. 1A), which was completely abolished in the Gαi1/3 DKO MEFs (Fig. 1A). Moreover, in WT MEFs RSPO3 (50 ng/mL) induced Akt/S6K/S6 phosphorylation in a time-dependent manner. It was however nullified in the Gαi1/3 DKO MEFs (Fig. 1A). Quantification results integrating five repeated blotting data showed that RSPO3 (50 ng/mL)-induced Akt/S6K/S6 phosphorylation was completely blocked in Gαi1/3 DKO MEFs (Fig. 1A). Expression of total Akt/S6K/S6 was comparable between WT and Gαi1/3 DKO MEFs (Fig. 1A). Result in the right panel confirmed depletion of Gαi1 and Gαi3, but not Gαi2, in the Gαi1/3 DKO MEFs (Fig. 1A).

**Figure 1.**
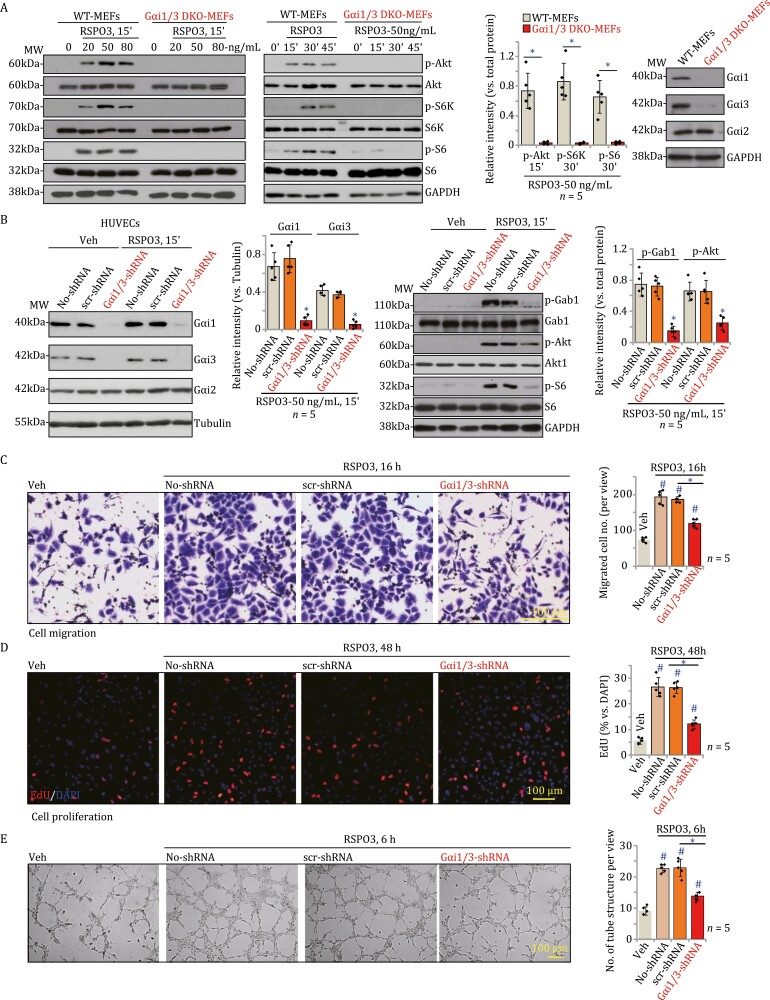
Gαi1/3 silencing inhibits RSPO3-induced Akt-mTOR activation and pro-angiogenic functions in cultured endothelial cells. (A) The wild-type (WT) or the Gαi1/3 double knockout (DKO) mouse embryonic fibroblasts (MEFs) were treated with the designated concentration of RSPO3 and cultivated for indicated time periods, expression of listed proteins was shown and protein phosphorylation was quantified. (B) Stable HUVECs expressing the lentiviral Gαi1 shRNA plus the lentiviral Gαi3 shRNA (“Gαi1/3-shRNA”) or the scramble control shRNA (“scr-shRNA”) were established, and were treated with RSOP3 (50 ng/mL) or the vehicle control (“Veh”). Cells were further cultured for the designated time periods, and expression of listed proteins was shown; (C) Cell migration (“Transwell” assays), (D) proliferation (by testing EdU-positive nuclei ratio) and (E) in vitro tube formation were tested by the listed assays. Data were presented as mean ± standard deviation (SD, *n* = 5). “MW” stands for molecular weight (same for all figures). “No-shRNA” stands for the parental control cells without shRNA infection. *P < 0.05 (A).*P < 0.05 versus “scr-shRNA” cells. ^#^*P* < 0.05 versus “Veh” treatment. The experiments were repeated five times with similar results obtained. Scale bar = 100 μm.

Next, we studied the individual role of Gαi1, Gαi2 and Gαi3 in RSPO3-induced Akt-mTOR activation in MEFs. Gαi1, Gαi2 or Gαi3 single knockout (“SKO”) MEFs were utilized [see the previous studies Cao et al. (2009), Zhang et al. (2015), Marshall et al. (2018), Sun et al. (2018), Bai et al. (2021), and Wang et al. (2021)]. RSPO3 (50 ng/mL)-induced Akt/S6K/S6 phosphorylation in Gαi1 SKO MEFs and Gαi3 SKO MEFs was relatively weak when compared to WT MEFs (Fig. S1A). Further quantification results supported that Gαi1 SKO or Gαi3 SKO in MEFs only partially inhibited RSPO3-induced Akt-mTOR activation, while Gαi1 and Gαi3 DKO almost completely abolished it (Fig. S1B). Figure S1C confirmed SKO of Gαi1 or Gαi3 in the corresponding MEFs, and Gαi2 expression was unchanged. RSPO3-induced Akt/S6K/S6 phosphorylation was equivalent between the WT MEFs and the Gαi2 SKO MEFs (Fig. S1D), indicating that Gαi2, unlike Gαi1 and Gαi3, might not be required for RSPO3-induced Akt-mTOR activation in MEFs.

To further support our hypothesis, the CRISPR/Cas9 gene editing method was employed to knockout (KO) Gαi1 and Gαi3 in MEFs. Single stable MEFs were established after KO screening and selection (see our previous studies Bai et al. [2021], and Wang et al. [2021]). These MEFs were named as CRISPR-Gαi1/3-DKO MEFs. As shown RSPO3 induced robust Akt/S6K phosphorylation in MEFs with the Cas9 control construct (“Cas9-C”) (Fig. S1E), it was however abolished in the CRISPR-Gαi1/3-DKO MEFs (Fig. S1E). Figure S1F confirmed Gαi1 and Gαi3 protein depletion in the CRISPR-Gαi1/3-DKO MEFs.

Next, shRNA method was utilized to silence Gαi1/3. Stable MEFs expressing the Gαi1 shRNA and the Gαi3 (“Gαi1/3-DshRNA”) or the scramble control shRNA (“scr-shRNA”) were described previously (Zhang et al. 2015; Marshall et al. 2018; Sun et al. 2018; Bai et al. 2021; Wang et al. 2021). As demonstrated, Gαi1/3-DshRNA remarkably inhibited RSPO3-induced Akt/S6K phosphorylation (Fig. S2A). Total Akt/S6K expression was again unchanged (Fig. S2A). Figure S2B confirmed robust Gαi1 and Gαi3 silencing by Gαi1/3-DshRNA (Zhang et al. 2015; Marshall et al. 2018; Sun et al. 2018; Bai et al. 2021; Wang et al. 2021). The latter failed to alter Gαi2 protein expression (Fig. S2B).

To the Gαi1/3 DKO MEFs the adenoviral murine Gαi1 expression construct (“Ad-Gαi1” [Marshall et al. 2018; Sun et al. 2018; Bai et al. 2021; Wang et al. 2021]) or the adenoviral murine Gαi3 expression construct (“Ad-Gαi3” [Marshall et al. 2018; Sun et al. 2018; Bai et al. 2021; Wang et al. 2021]) was transduced, and stable cells were established after selection. Figure S2C showed that Ad-Gαi1 or Ad-Gαi3 partially restored RSPO3-induced Akt/S6K phosphorylation in Gαi1/3 DKO MEFs. The rescue experiment results and the SKO results supported that both Gαi1 and Gαi3 are required for RSPO3-induced Akt-mTOR activation in MEFs. Figure S2D confirmed restoring Gαi1 or Gαi3 protein expression by Ad-Gαi1 or Ad-Gαi3 in Gαi1/3 DKO MEFs. Gαi2 protein expression was again unchanged (Fig. S2D).

RSPO3 is known to activate and amplify Wnt/β-catenin signaling through different mechanisms. Expression of the key proteins in RSPO3-Wnt/β-catenin cascade, including LGR4, β-catenin, RNF43, Disheveled (DVL) and frizzled (FZD), was indifferent between WT and Gαi1/3 DKO MEFs (Fig. S3A). Our previous studies have found that Gαi1/3 can directly associate with cell-surface receptors by different stimuli, mediating downstream signaling transduction (Marshall et al. 2018; Sun et al. 2018; Bai et al. 2021; Wang et al. 2021; Bian et al. 2022). The co-immunoprecipitation (“Co-IP”) assay results discovered that following RSOP3 treatment Gαi1/3 associated with LGR4 and Grb2-associated binder 1 (Gab1) in WT MEFs (Fig. S3B). Gab1 is a key adaptor protein required for Akt-mTOR activation by growth factors and various stimuli. RNF43 and DVL were not immunoprecipitated with Gαi1/3 and Gab1 in RSOP3-treated MEFs (Fig. S3B). “Input” control results showed that treatment with RSOP3 failed to significantly alter expression of these signaling proteins (LGR4, Gαi1/3, Gab1, DVL and RNF43) in MEFs (Fig. S3B).

Next experiments were carried out to explore the potential effect of the LGR4-Gαi1/3-Gab1 complex in RSOP3-induced Akt-mTOR activation. The lentiviral constructs encoding two different LGR4 shRNAs, sh-LGR4-s1 and sh-LGR4-s2, were individually tranduced to WT MEFs, stable cells were formed following selection using the puromycin containing medium. The two shRNAs resulted in robust LGR4 protein silencing in WT MEFs, without affecting Gαi1 and Gαi3 protein expression (Fig. S3C). Importantly, LGR4 silencing almost blocked RSOP3-induced Akt activation in MEFs (Fig. S3C).

To examine whether Gab1 was required for RSPO3-induced Akt-mTOR activation, WT MEFs and Gab1 KO MEFs (Cao et al. 2009; Zhang et al. 2015; Bai et al. 2021) were utilized. Figure S3D showed that RSPO3 activated Gab1 and induced Gab1 phosphorylation (at Tyr-627) in WT MEFs. Importantly, RSPO3-induced Akt/S6 phosphorylation was almost completely blocked in Gab1 KO MEFs (Fig. S3D). These results supported that Gab1, in association with Gαi1 and Gαi3, was required for RSPO3-induced Akt-mTOR activation. Exploring the relationship between Gab1 and Gαi1/3 in mediating Akt-mTOR activation by RSPO3, we showed that Gαi1/3 should be the upstream signaling proteins for RSPO3-induced Gab1 activation. In both Gαi1/3 DKO MEFs and CRISPR-Gαi1/3-DKO MEFs, RSPO3-induced Gab1 phosphorylation was completely abolished (Fig. S3E). Whereas Gαi1 SKO or Gαi3 SKO, but not Gαi2 SKO, partially inhibited RSPO3-induced Gab1 activation (Fig. S3F). Moreover, RSPO3-induced Gab1 phosphorylation was largely inhibited by Gαi1/3 DshRNA (Fig. S3G), but was augmented following Gαi1/3 overexpression (“OE-Gαi1/3”, Fig. S3H). These results together supported that Gab1, the downstream signaling adaptor protein of LGR4-Gαi1/3, was required for RSPO3-induced Akt-mTOR activation in MEFs. RSPO3-induced active β-Catenin accumulation was unaffected in both Gαi1/3 DKO MEFs and CRISPR-Gαi1/3-DKO MEFs (Fig. S3I).

To block Gαi1/3 association with other signaling proteins, the dominant negative (“DN”) mutants of Gαi1 and Gαi3 were transduced into WT MEFs (“DN-Gαi1/3”). These Gαi1/3 mutants replace the conserved Gly (G) residue with Thr (T) in G3 box preventing Gαi1/3 association with adaptor/associated proteins (see the previous studies Cao et al. [2009], and Zhang et al. [2015]). In DN-Gαi1/3-expressing MEFs, RSPO3 (50 ng/mL, 5 min)-induced LGR4-Gαi1/3-Gab1 association was completely blocked (Fig. S4A and S4B). DN-Gαi1/3 largely inhibited RSPO3-induced Akt/S6K/S6 phosphorylation in MEFs (Fig. S4C). Figure S4D confirmed expression of DN-Gαi1 and DN-Gαi3 in the MEFs. These results further supported that RSOP3 induced Gαi1/3 association with LGR4 and Gab1, mediating downstream Akt-mTOR activation.

Next we tested whether Gαi1/3 proteins were required for RSPO3-induced Akt-mTOR activation in endothelial cells. In cultured human umbilical vein endothelial cells (HUVECs) (Sun et al. 2018; Yao et al. 2022), Gαi1 shRNA lentivirus and Gαi3 shRNA lentivirus (Sun et al. 2018; Wang et al. 2021) were both added. After puromycin selection, the stable HUVECs expressing both shRNA (“Gαi1/3-shRNA”) were formed. Control HUVECs were stably transduced with scramble control shRNA (“scr-shRNA”). As shown Gαi1 and Gαi3 protein expression was remarkably decreased in Gαi1/3-shRNA HUVECs (Fig. 1B), where Gαi2 protein expression was unchanged (Fig. 1B). Importantly, Gαi1/3-shRNA potently inhibited RSPO3 (50 ng/mL, 15ʹ)-induced phosphorylation of Gab1 and Akt/S6 in HUVECs (Fig. 1B). Total Gab1 and Akt/S6 protein expression was unaffected by Gαi1/3-shRNA (Fig. 1B).

Gαi1 and Gαi3 double silencing in HUVECs largely inhibited RSPO3-induced cell migration (Fig. 1C) and proliferation (Fig. 1D), which were tested by “Transwell” (Fig. 1C) and nuclear 5-ethynyl-2ʹ-deoxyuridine (EdU) staining (Fig. 1D) assays, respectively. RSPO3 significantly increased the number of the tube-like structures in HUVECs, which was inhibited by Gαi1/3-shRNA (Fig. 1E). Notably, Gαi1/3-shRNA inhibited, but not reversed, RSPO3-induced pro-angiogenic response in HUVECs (Fig. 1C–E).

The Gαi1 shRNA lentivirus and the Gαi3 shRNA lentivirus were also employed to stably knockdown Gαi1 and Gαi3 in the hCMEC/D3 brain endothelial cells (“Gαi1/3-shRNA”). shRNA-induced silencing of Gαi1 and Gαi3 almost completely blocked RSPO3 (50 ng/mL, 15ʹ)-induced Gab1 and Akt/S6K/S6 phosphorylation in hCMEC/D3 cells (Fig. S5A and S5B). Gαi1/3-shRNA inhibited RSPO3-induced cell migration (Fig. S5C) and proliferation (Fig. S5D) in hCMEC/D3 endothelial cells. Thus Gαi1/3 are essential for RSPO3-induced Akt-mTOR activation and pro-angiogenic activity in endothelial cells.

If Gαi1/3 mediation of RSPO3-induced pro-angiogenic functions is due to promoting Akt-mTOR activation, restoring Akt-mTOR activation should reverse Gαi1/3-shRNA-induced inhibitory actions on angiogenesis. Therefore, a constitutively-active Akt1 (caAkt1, S473D [Yao et al. 2022]) construct was transduced to Gαi1/3-shRNA-expressing HUVECs. As shown caAkt1 failed to affect Gαi1/2/3 expression (Fig. S6A), but it completely restored Akt/S6K phosphorylation in Gαi1/3-silenced HUVECs (“Gαi1/3-shRNA”, Fig. S6B). With caAkt1 rescuing Akt-mTOR activation, RSPO3-induced cell migration (Fig. S6C) and proliferation (Fig. S6D) were completely restored in Gαi1/3-shRNA HUVECs.

Next, the adenoviral Gαi1 expressing construct and the adenoviral Gαi3 expressing construct were co-tranduced to HUVECs, and the stable cells were established following selection (“OE-Gαi1/3”, Fig. S6E). Gαi1 and Gαi3, but not Gαi2, were significantly upregulated in OE-Gαi1/3 HUVECs (Fig. S6E). Consequently, RSPO3 (50 ng/mL, 15ʹ)-induced phosphorylation of Gab1 and Akt/S6K was remarkably increased (Fig. S6F). RSPO3-induced cell migration and proliferation (EdU-nuclei percentage) were augmented in OE-Gαi1/3 HUVECs (Fig. S6G). Contrarily, treatment with LY294002, the Akt-mTOR blocker, largely inhibited RSPO3-induced migration and proliferation in Gαi1/3-overexpressed HUVECs (Fig. S6H and S6I).

To the hCMEC/D3 brain endothelial cells, the adenoviral Gαi1 expressing construct and the adenoviral Gαi3 expressing construct were co-transduced. Stable cells, namely OE-Gαi1/3 hCMEC/D3 cells, were established. Gαi1 and Gαi3 expression as well as RSPO3-induced Gab1 and Akt/S6 phosphorylation were robustly increased in OE-Gαi1/3 hCMEC/D3 cells (Fig. S7A and S7B). Moreover, Gαi1/3 overexpression further enhanced RSPO3-induced cell migration (Fig. S7C) and proliferation (EdU-nuclei percentage, Fig. S7D) in hCMEC/D3 cells.

To further investigate the role of Gαi1/3 in RSPO3-induced angiogenesis *in vivo*, C57B/6 mice were intra-vitreously injected with the AAV5-RSPO3 expression construct containing the endothelial cell-specific promoter TIE1 (reported in our previous study Yao et al. [2022], Fig. 2A): causing endothelial RSPO3 over-expression (“eOE-RSPO3”) (Fig. 2A). In addition, mice were intravitreously co-injected with the AAV5-TIE1-Gαi1 shRNA plus the AAV5-TIE1-Gαi3 shRNA, leading to endothelial Gαi1/3 knockdown (“Gαi1/3-eKD”) (Fig. 2A). The control group mice were intravitreously injected with the AAV5-TIE1-scramble control shRNA (“Ctrl”) (Yao et al. 2022).

**Figure 2.**
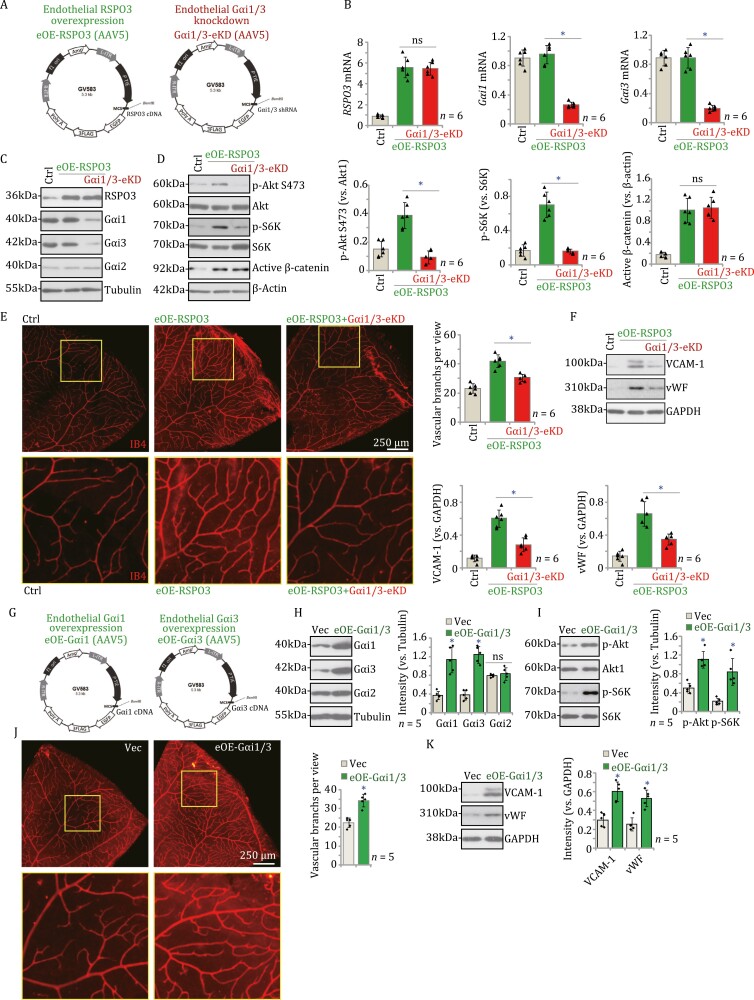
Gαi1/3 are important for RSPO3-induced Akt-mTOR activation and retinal angiogenesis *in vivo*. (A) The C57B/6 adult mice (4-week, all male) were intra-vitreously injected with the AAV5-TIE1-RSPO3 expression construct (“eOE-RSPO3”), with or without the AAV5-TIE1-Gαi1 shRNA construct plus the AAV5-TIE1-Gαi3 shRNA construct (“Gαi1/3-eKD”). (B–D and F) Control mice were intra-vitreously injected with the AAV5-TIE1-scramble control shRNA (“Ctrl”). After 10 days, expression of listed mRNAs and proteins in the retinal tissues was tested. (E) The retinal vasculature was measured by IB4 staining and the average number of vascular branches per view was calculated. (G) The C57B/6 adult mice (4-week, all male) were intra-vitreously injected with the the AAV5-TIE1-Gαi1 expression construct plus the AAV5-TIE1-Gαi3 expression construct (“eOE-Gαi1/3”). Control mice were intra-vitreously injected with AAV5-TIE1-empty vector (“Vec”). (H, I and K) After 10 days, expression of listed proteins in the retinal tissues was tested. (J) The retinal vasculature was measured by IB4 staining and the average number of vascular branches per view was calculated (J). The data were presented as mean ± standard deviation (SD, *n* = 6). *P < 0.05 (B–F). *P < 0.05 versus “Vec” group (H–K). “ns” stands for non-statistical differences (*P* > 0.05). The experiments were repeated five to six times with similar results obtained. Scale bar = 250 μm.

Ten days after virus injection, the retinal tissues were homogenized and were analyzed by qRT-PCR and Western blotting assays. In the retinal tissues of the eOE-RSPO3 mice, *RSPO3* mRNA and protein expression was significantly increased (Fig. 2B and 2C), whereas mRNA and protein expression levels of Gαi1 and Gαi3 were not significantly altered (Fig. 2B and 2C). Importantly, eOE-RSPO3 increased Akt-S6K phosphorylation and augmented active β-catenin contents (Fig. 2D). The retinal isolectin B4 (IB4) staining assay results demonstrated that endothelial over-expression of RSPO3, eOE-RSPO3, resulted in remarkable increase in retinal angiogenesis. The eOE-RSPO3 retinas displayed increased number of vascular branches and branch points, and enhanced retinal vascular complexity (Fig. 2E).

In the eOE-RSPO3 mice, further Gαi1/3-eKD (Fig. 2A) silenced Gαi1 and Gαi3, without affecting Gαi2 and RSPO3 expression in the retinal tissues (Fig. 2B and 2C). Remarkably, eOE-RSPO3-induced Akt-S6K phosphorylation was largely inhibited by Gαi1/3-eKD (Fig. 2C). These results were in line with the *in vitro* findings. eOE-RSPO3-induced upregulation of active β-catenin in the retinal tissues was not affected by endothelial Gαi1/3 silencing (Fig. 2D). Importantly, eOE-RSPO3-promoted retinal angiogenesis was largely inhibited by Gαi1/3-eKD (Fig. 2E). Moreover, expression endothelial marker proteins, vascular cell adhesion molecule-1 (VCAM-1) and von willebrand factor (vWF), was significantly increased in retinal tissues of eOE-RSPO3 mice (Fig. 2F), which was again inhibited after endothelial Gαi1/3 silencing (Fig. 2F). These results further supported that Gαi1/3 are important for RSPO3-induced Akt-mTOR activation and retinal angiogenesis *in vivo*.

We further hypothesized that endothelial overexpression of Gαi1 and Gαi3 could enhance RSPO3 signaling and angiogenesis. Therefore, the AAV5-TIE1-Gαi1 expression construct (Fig. 2G) and the AAV5-TIE1-Gαi3 expression construct (Fig. 2G) were intravitreously injected to the C57B/6 mice, aiming to induce endothelial Gαi1 and Gαi3 overexpression (“eOE-Gαi1/3”). The control group mice were intravitreously injected with the AAV5-TIE1 empty vector (“Vec”). Ten days after virus injection, the retinal tissues were homogenized and analyzed. As shown, in eOE-Gαi1/3 retinal tissues, protein levels of Gαi1 and Gαi3, but not Gαi2, were significantly upregulated (Fig. 2H). Increased Akt-mTOR activation was detected in eOE-Gαi1/3 retinal tissues, as Akt-S6K phosphorylation was remarkably enhanced (Fig. 2I). The number of vascular branches and branch points, as well as the retinal vascular complexity were significantly increased in eOE-Gαi1/3 retinas (Fig. 2J). VCAM-1 and vWF expression was upregulated as well (Fig. 2K). Together, these results supported that endothelial Gαi1/3 overexpression promoted retinal angiogenesis *in vivo*.

RSPO3 expression in highly vascularized tissues and the vascular phenotype of RSPO3-KO or mutant mice supported a primary role of RSPO3 in endothelial cells. Activation and amplification of Wnt/β-catenin signaling is important for RSPO3-induced vascular development and angiogenesis (Kazanskaya et al. 2008). However it is possible that other cascades could also participate in the process. Indeed, multiple reports confirmed significant Akt cascade activation by RSPO3 in cancerous cells (Chen et al. 2020; Gu et al. 2020). Here we discovered that RSPO3 activated Wnt/β-catenin-independent Akt-mTOR signaling in endothelial cells, which was important for angiogenesis.

We have previously shown that Gαi1/3 are key signaling proteins mediating Akt-mTOR cascade activation by various receptor tyrosine kinase ligands, including epidermal growth factor (EGF) (Cao et al. 2009), keratinocyte growth factor (KGF) (Zhang et al. 2015), brain-derived neurotrophic factor (BDNF) (Marshall et al. 2018). Recently, we found that Gαi1/3 immunoprecipitated with neuroligin 3-activated receptor tyrosine kinases, mediating downstream Akt-mTOR activation and glioma cell growth *in vitro* and *in vivo* (Wang et al. 2021). Following interleukin 4 (IL4) stimulation, Gαi1/3 associated with the intracellular domain of IL-4Rα, promoting IL-4Rα endosomal traffic and downstream Akt activation in macrophages. Gαi1/3 silencing or KO inhibited IL-4-induced Akt-mTOR activation and macrophage M2 polarization (Bai et al. 2021). These results supported the key role of Gαi1/3 in mediating Akt-mTOR signaling under different stimuli.

The results of the present study supported that Gαi1/3 are key signaling proteins required for RSPO3-induced Akt-mTOR activation. In MEFs, RSPO3-induced Akt-mTOR activation was completely abolished following Gαi1/3 DKO. Gαi1 or Gαi3 SKO only partially attenuated RSPO3-induced Akt-mTOR activation in MEFs; While Gαi2 SKO was completely ineffective. Moreover, Gαi1/3 silencing or CRISPR/Cas9-induced Gαi1/3 DKO remarkably inhibited Akt-mTOR activation by RSPO3 in MEFs. Importantly, exogenous expression of Gαi1 or Gαi3 in Gαi1/3 DKO MEFs partially restored RSPO3-induced Akt-mTOR activation. In HUVEC and HCMEC/D3 RSPO3-induced Akt-mTOR activation was largely inhibited by Gαi1/3 silencing, but was augmented with ectopic Gαi1/3 overexpression. Therefore, Gαi1/3 are required for RSPO3-induced Akt-mTOR activation in endothelial cells.

Here we showed that RSOP3 induced Gαi1/3 association with LGR4 and Gab1, required for the downstream Akt-mTOR activation. RSPO3-induced Akt-mTOR activation was largely inhibited by LGR4 silencing or Gab1 KO. Gab1 should be primary downstream signaling protein of Gαi1/3 in mediating RSPO3-induced Akt-mTOR activation. As Gαi1/3 shRNA or KO largely inhibited RSPO3-induced Gab1 phosphorylation, whereas Gαi1/3 overexpression enhanced it. Importantly, Gab1 KO blocked RSPO3-induced Akt-mTOR activation in MEFs. Therefore, RSOP3 induced LGR4-Gαi1/3-Gab1 signaling complex formation, mediating downstream Akt-mTOR activation. Gαi1/3 silencing failed to affect RSPO3-induced active β-catenin accumulation in MEFs and HUVECs.

We have previously shown that Gαi1/3 are important signaling proteins regulating endothelial cell functions and angiogenesis. We showed that under VEGF stimulation Gαi1/3 association with the VEGFR2 endocytosis complex (VEGFR2-Ephrin-B2-Dab2-PAR-3) promoted VEGFR2 endocytosis, downstream Akt-mTOR transduction and angiogenesis (Sun et al. 2018). Recently, we found that phosphoenolpyruvate carboxykinase 1 (PCK1) was required for angiogenesis *in vitro* and *in vivo*, possibly by promoting Gαi3 expression and Akt-mTOR activation (Yao et al. 2022).

Here we found that in both HUVECs and hCMEC/D3 cells, shRNA-induced silencing of Gαi1/3 remarkably inhibited RSPO3-induced cell migration, proliferation, and *in vitro* tube formation. Whereas ectopic overexpression of Gαi1/3 exerted opposite effects and augmented RSPO3-induced pro-angiogenesis response *in vitro*. Importantly, caAkt1 restored Akt-mTOR activation in Gαi1/3-silenced HUVECs, and it also recovered RSPO3-induced pro-angiogenesis activity *in vitro*. *In vivo*, conditional knockdown of Gαi1/3 in endothelial cells significantly inhibited endothelial RSPO3 overexpression-induced Akt-mTOR activation and retinal angiogenesis in mice. Contrarily, endothelial overexpression of Gαi1/3 increased Akt-mTOR activation and retinal angiogenesis *in vivo*. Together, our results supported that Gαi1/3 mediation of Akt-mTOR cascade activation is important for RSPO3-induced angiogenesis *in vitro* and *in vivo*.

## Supplementary information

The online version contains supplementary material available at https://doi.org/10.1093/procel/pwac035.

pwac035_suppl_Supplementary_Material

## Footnotes

This work was generously supported by Key Research and Development Program of Jiangsu Province (No. BE2019652) and National Natural Science Foundation of China (81922025, 81974388, 82171461, 81771457). A Project Funded by the Priority Academic Program Development of Jiangsu Higher Education Institutions.

The authors declare that they have no competing interests.

This study was approved by Ethics Committee of Soochow University. All institutional and national guidelines for the care were carefully followed.

All data generated or analyzed during this study are included in this published article (and its supplementary information file).

All authors conceived, designed, and supervised the study. All authors collected samples, performed the experiments, analyzed the data, and involved in drafting the article and revising it critically for important intellectual content.
