# Supplementary material for: Gαi1/3 mediation of Akt-mTOR activation is important for RSPO3-induced angiogenesis
Source: Protein Cell. 2022 Aug 12;14(3):217–22. doi: 10.1093/procel/pwac035 (PMC10098032; doi:10.1093/procel/pwac035)
Supplement: pwac035_suppl_Supplementary_Material [file pwac035_suppl_supplementary_material.pdf]

Supplementary information

Gαi1/3 mediation of Akt-mTOR activation is important for RSPO3-induced angiogenesis

Gang Xu [1, 2#], Li-na Qi [1#], Mei-qing Zhang [1#], Xue-yun Li [1], Jin-long Chai [1], Zhi-qing Zhang [1], Xia Chen [3], Qian Wang [3*], Ke-ran Li [4*], and Cong Cao [1, 4*]

This file includes:
Materials and methods
Figures S1-S7

Materials and methods

Reagents. Cell culture reagents were provided by Gibco (Suzhou, China). The Gαi1/3-Gab1-Akt-mTOR antibodies were reported previously (Liu et al., 2018). Other antibodies were from Cellular Signaling Tech (Suzhou, China) and Abcam (Beijing, China). RSPO3 and LY294002 were provided by Sigma-Aldrich (St. Louis, Mo).

Cell culture. Culturing of the wild-type (WT), Gαi1/3 double knockout (DKO), Gαi1, Gαi2 and Gαi3 single knockout (SKO) mouse embryonic fibroblasts (MEFs) as well as WT and Gab1 KO MEFs was described in our previous studies (Cao et al., 2009; Li et al., 2015; Zhang et al., 2015; Marshall et al., 2018; Sun et al., 2018; Bai et al., 2021; Wang et al., 2021). Culturing of the human umbilical vein endothelial cells (HUVECs) and hCMEC/D3 brain endothelial cells were also described previously (Sun et al., 2018; Zhang et al., 2018; Yao et al., 2022).

Genetic modifications. Stable MEFs with the lentiviral mouse Gαi1 shRNA plus the lentiviral mouse Gαi3 shRNA ("Gαi1/3 DshRNA") or scramble control shRNA ("scr-shRNA"), the adenoviral mouse Gαi1 expression construct ("Ad-Gαi1") plus the adenoviral Gαi3 expression construct ("Ad-Gαi3"), the dominant negative (DN)-Gαi1 or DN-Gαi3, or the empty vector ("Vec") were described in our previous studies (Cao et al., 2009; Zhang et al., 2015; Marshall et al., 2018; Sun et al., 2018; Bai et al., 2021; Wang et al., 2021).CRISPR/Cas9-induced Gαi1 and Gαi3 double knockout (CRISPR/Cas9-Gαi1/3-DKO) in MEFs was reported (Sun et al., 2018; Bai et al., 2021; Wang et al., 2021). For the rescue experiments, the Ad-Gαi1 or the Ad-Gαi3 were tranduced to Gαi1/3 DKO MEFs (Marshall et al., 2018; Sun et al., 2018; Bai et al., 2021; Wang et al., 2021). HUVECs or hCMEC/D3 were infected with the human Gαi1 shRNA-expressing lentivirus plus human Gαi3 shRNA-expressing lentivirus ("Gαi1/3 DshRNA", see (Sun et al., 2018; Bai et al., 2021; Bian et al., 2022)) or scramble control shRNA ("scr-shRNA"), and stable cells were formed after adding puromycin selection medium for five-six passages (Sun et al., 2018). Alternatively HUVECs or hCMEC/D3 were transduced with the adenoviral human Gαi1 expression construct ("Ad-Gαi1") plus the adenoviral human Gαi3 expression construct ("Ad-Gαi3") or the empty vector ("Vec") (Wang et al., 2021; Bian et al., 2022), and stable cells were formed after adding puromycin selection medium for five-six passages. For gene silencing or overexpression *in vivo*, Gαi1/Gαi3 shRNA sequence, Gαi1/Gαi3 expression sequence, or RSPO3 expression sequence was inserted into an adeno-associated virus 5 (AAV5)-TIE1 construct (reported in our previous study (Yao et al., 2022)) that contained

sequence of the endothelial specific promoter TIE1 (Yao et al., 2022). The constructs were individually transfected to HEK-293 cells to generate adenovirus, which was intravitreally injected to the mice as reported (Yao et al., 2022). The GV369 constructs containing the LGR4 shRNA (sh-LGR4-s1 or sh-LGR4-s2) were provided by Genechem (Shanghai, China). The constructs were individually transfected to HEK-293 cells to generate lentivirus. Virus were thereafter enriched, purified and quantified, and were added to cultured cells. Stable cells were formed after selection using puromycin containing medium.

Constitutively-active mutant Akt1. The recombinant adenoviral constitutively-active Akt1 (caAkt1, S473D) construct was described in our previous study (Bian et al., 2022) and was transduced to HUVECs. Stable cells were formed by adding puromycin selection medium and cells were then distributed to 96-well plates. The caAkt1 expression in single stable cells was verified by Western blotting.

Other assays, including Western blotting, co-immunoprecipitation (Co-IP), quantitative real-time PCR (qRT-PCR), the nuclear EdU (5-ethynyl-2′-deoxyuridine)/DAPI (4',6-diamidino-2-phenylindole) staining, “Transwell” and *in vitro* tube formation assays were described in our previous studies (Sun et al., 2018; Lv et al., 2021; Wang et al., 2021; Yao et al., 2022).

Intravitreal injection of AAV and retinal endothelial isolectin B4 (IB4) staining. The adult C57BL/6 mice were provided by SLAC Laboratory Animal Center (Shanghai, China) and maintained as reported (Yao et al., 2022). Mice were anesthetized using the previously-described method (Yao et al., 2022). A 33-gauge disposable needle was utilized to inject AAV to vitreous cavity as reported (Yao et al., 2022). For IB4 staining, the eyeballs were removed and fixed in ice-cold 4% paraformaldehyde for 30 min. The retinas were carefully isolated and placed on the glass slide, cut into four-leaf clover shape and were fixed by 4% paraformaldehyde for 15-20 min. The retina was washed and was blocked by 1% Triton X-100+3% BSA (in PBS) at 37°C for 45 min. After aspirating the blocking solution, the retinas were stained with IB4 (1:50) at 4°C for 12h. IB4 staining was visualized under a confocal microscope. The animal protocols were conducted in according to the Institutional Animal Care and Use Committee and the Ethic Committee of Soochow University, and were complied with the provision of the ARVO (Association for Research in Vision and Ophthalmology) statement.

Statistical analyses. Data in this study were all with normal distribution and were always expressed as means ± standard deviation (SD). To examine differences between three or more groups, one-way ANOVA and the Scheffe's f-test (SPSS 23.0, SPSS Co., Chicago, CA) were utilized. The two-tailed unpaired t test (Excel 2007) was utilized when examining difference between two groups. ***P*** values < 0.05 were considered statistically significant.

Figure S1.

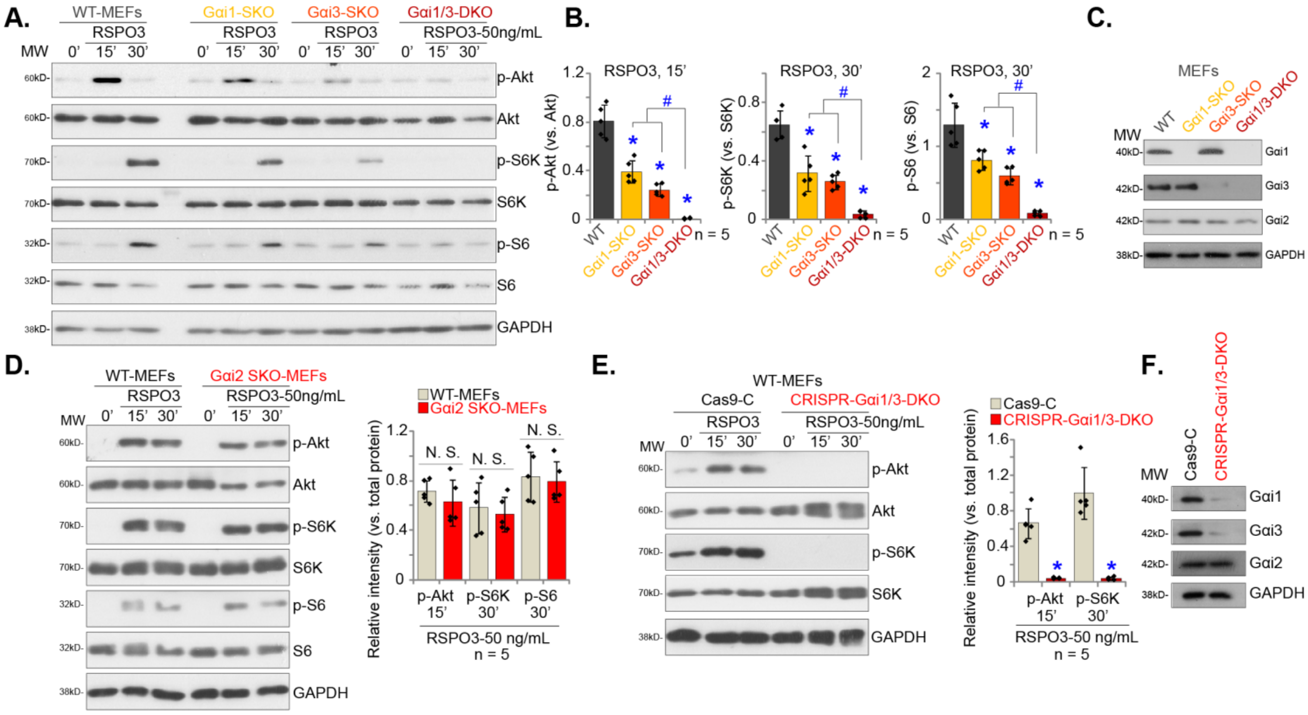

Figure S1. Gαi1 and Gαi3 knockout inhibits RSPO3-induced Akt-mTOR activation in mouse embryonic fibroblasts (MEFs). The wild-type (WT), Gαi1, Gαi2 and Gαi3 single knockout (SKO) mouse embryonic fibroblasts (MEFs) were treated with the designated concentration of RSPO3 and cultivated for indicated time periods, expression of listed proteins was shown and protein phosphorylation was quantified (**A**-**D**). WT MEFs stably expressing the CRISPR-Gαi1-KO construct plus the CRISPR-Gαi3-KO construct ("CRISPR-Gαi1/3-DKO") or the Cas9 control construct ("Cas9-C") were treated with RSPO3 (50 ng/mL) and cultured for 15-45 min, expression of listed proteins was shown and protein phosphorylation was quantified (**E** and **F**). "MW" stands for molecular weight (Same for all Figures). Data were expressed as mean ± standard deviation (SD). Quantifications were from five replicate blot data (n = 5). *P < 0.05 versus "WT MEFs" or "Cas9-C" treatment. # P < 0.05. "N. S." stands for non-statistical differences (P > 0.05) (**D**).

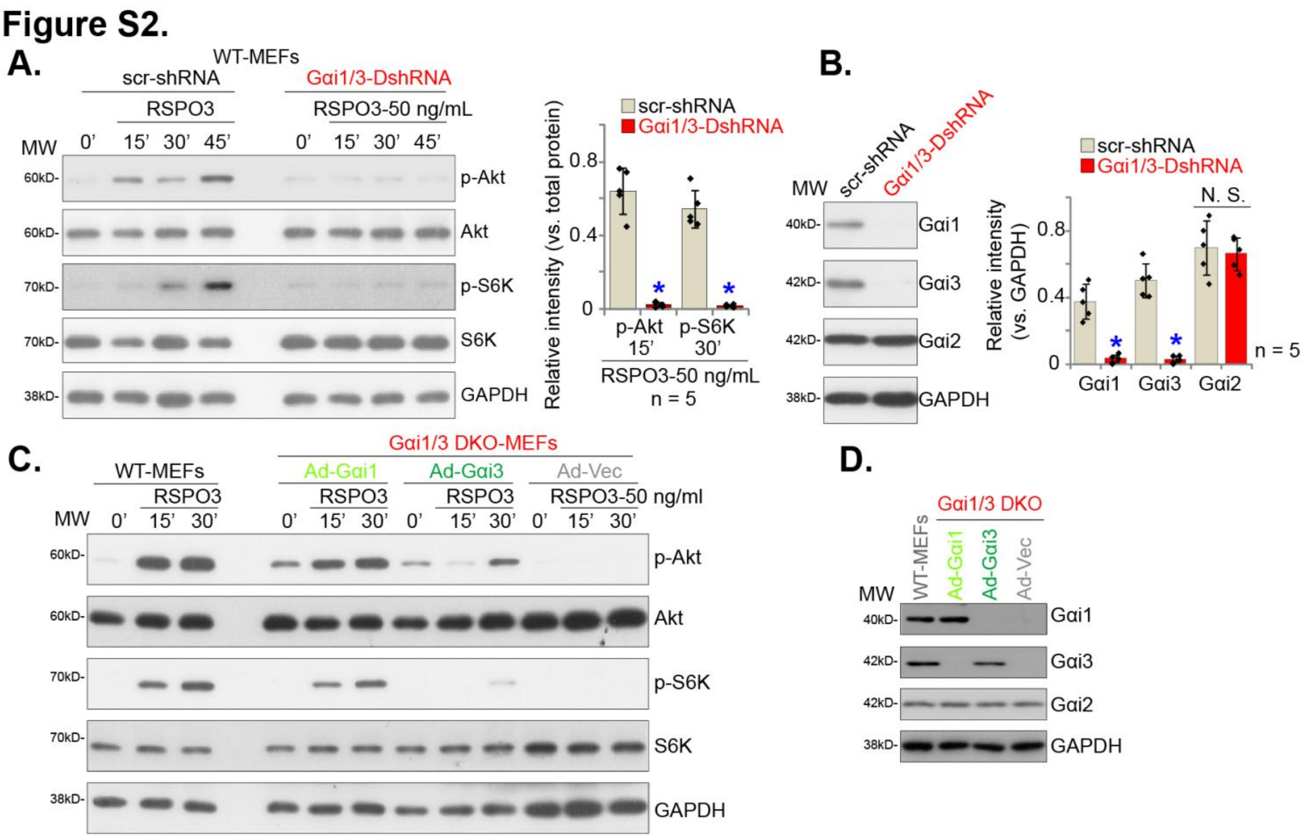

Figure S2. Gαi1 and Gαi3 are key signaling proteins for RSPO3-induced Akt-mTOR activation in MEFs. Stable MEFs with the Gαi1 shRNA plus the Gαi3 shRNA ("Gαi1/3-DshRNA") or the scramble control shRNA ("scr-shRNA") were treated with RSPO3 (50 ng/mL) and cultured for 15-45 min, expression of listed proteins was shown (**A** and **B**). Gαi1/3 DKO MEFs were stably transduced with the adenoviral mouse Gαi1 expression construct ("Ad-Gαi1"), the adenoviral mouse Gαi3 expression construct ("Ad-Gαi3") or the empty vector ("Ad-Vec"), treated with RSPO3 (50 ng/mL) and cultured for 15-45 min, expression of listed proteins was shown (**C** and **D**). Data were expressed as mean ± standard deviation (SD). Quantifications were from five replicate blot data (n = 5). ******P* < 0.05. versus "scr-shRNA" treatment. "N. S." stands for non-statistical differences (*P* > 0.05) (**B**).

Figure S3.

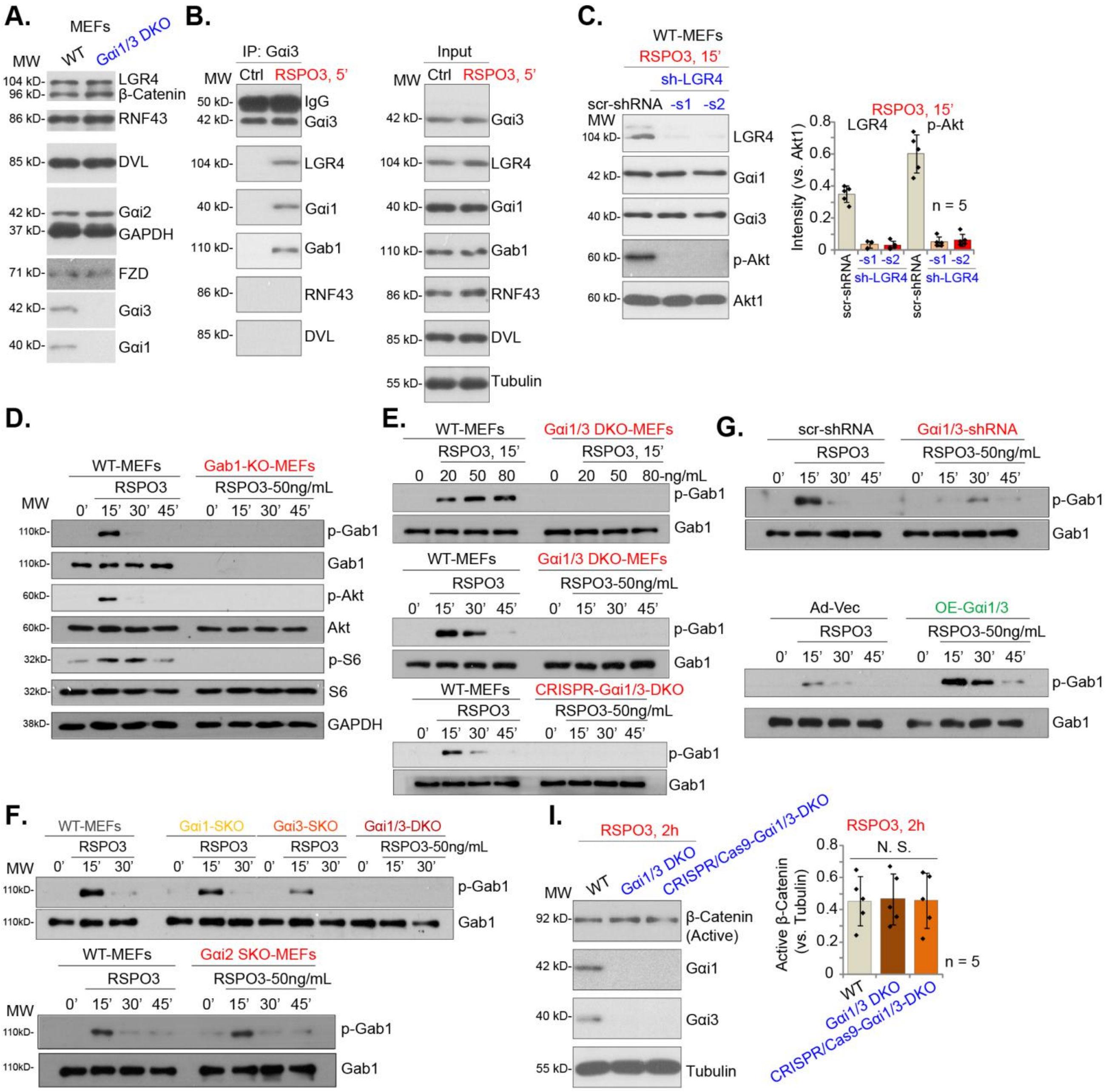

Figure S3. RSOP3 induces Gαi1/3 association with LGR4 and Gab1, required for downstream Akt-mTOR activation. Expression of the listed proteins in wild-type (WT) MEFs and Gαi1/3 double knockout (DKO) MEFs was shown (**A**); WT MEFs were treated with RSPO3 (50 ng/mL) and cultured for 5 min, LGR4-Gαi1/3-Gab1 association was tested by co-immunoprecipitation (Co-IP) assays (**B**, "IP"), expression of the listed proteins in the total cell lysates was tested by Western blotting assays (**B**, "Input"). WT MEFs stably expressing the lentiviral LGR4 shRNA (sh-LGR4-s1 and sh-LGR4-s2, representing two different sequences) or the scramble control shRNA ("scr-shRNA") were treated with RSPO3 (50 ng/mL) and cultured for 15 min, expression of listed proteins was tested (**C**).WT, Gab1 knockout (Gab1 KO),

Gαi1/3 DKO, Gαi1, Gαi2 and Gαi3 single knockout (SKO) MEFs, WT MEFs with the CRISPR-Gαi1-KO construct and the CRISPR-Gαi3-KO construct (CRISPR-Gαi1/3-DKO), the Cas9 control construct ("Cas9-C"), Gαi1 shRNA plus the Gαi3 ("Gαi1/3-DshRNA") or the scramble control shRNA ("scr-shRNA") were treated with RSPO3 (50 ng/mL) and cultured for 15-45 min, expression of listed proteins was shown (**D-G**, **I**). WT MEFs or Gαi1/3 DKO MEFs were stably transduced with the adenoviral mouse Gαi1 expression construct ("Ad-Gαi1"), the adenoviral mouse Gαi3 expression construct ("Ad-Gαi3") or the empty vector ("Ad-Vec"), and were treated with RSPO3 (50 ng/mL) and cultured for 15-45 min, expression of listed proteins was shown (**H**). Data were expressed as mean ± standard deviation (SD). Quantifications were from five replicate blot data (n = 5). ***P** < 0.05. versus "scr-shRNA" treatment (**C**). "N. S." stands for non-statistical differences (***P*** > 0.05).

Figure S4.

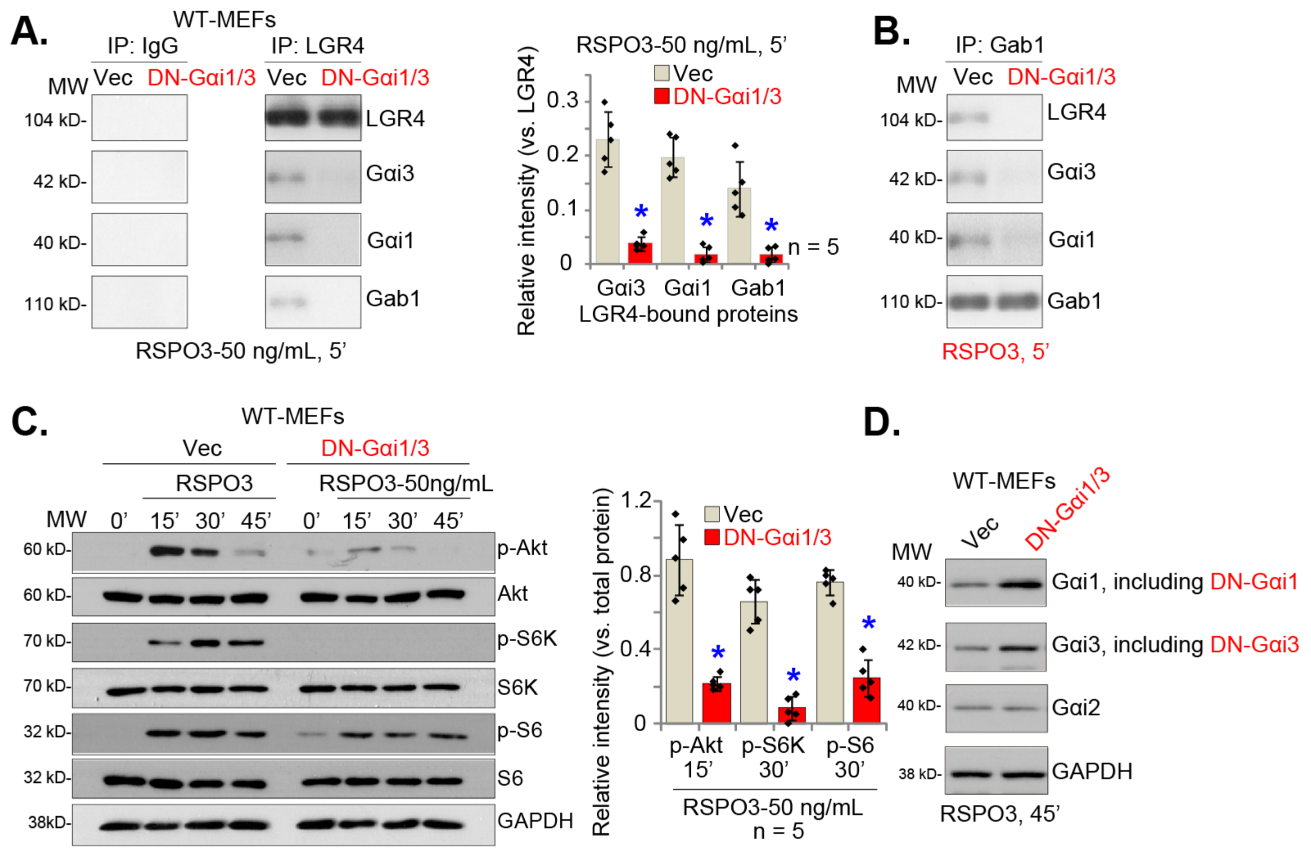

Figure S4. Gαi1/3 dominant negative mutation inhibits RSPO3-induced Akt-mTOR activation in MEFs. WT MEFs with the empty vector (“Vec”) or the dominant negative Gαi1 construct plus dominant negative Gαi3 construct (“DN-Gαi1/3”) were treated with RSPO3 (50 ng/mL) and cultured for 5 min, LGR4-Gαi1/3-Gab1 association was tested by co-immunoprecipitation (Co-IP) assays (**A** and **B**). MEFs were also treated with RSPO3 (50 ng/mL) for indicated time periods, expression of listed proteins was examined (**C** and **D**). Data were expressed as mean ± standard deviation (SD). Quantifications were from five replicate blot data (n = 5). *$P < 0.05$. versus “Vec” treatment (**C**).

Figure S5.

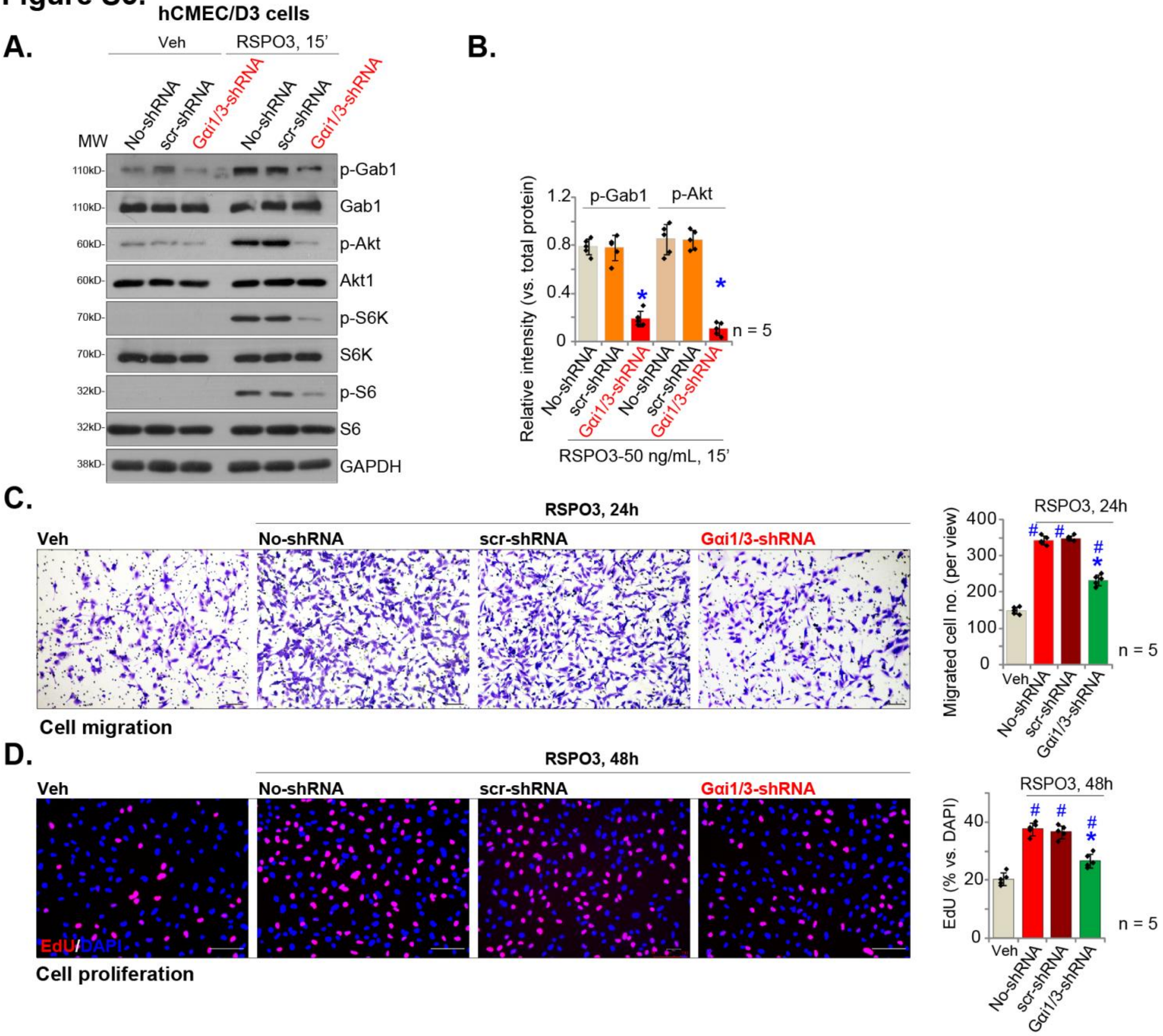

Figure S5. **Gαi1/3 silencing inhibits RSPO3-induced Akt-mTOR activation and pro-angiogenic functions in hCMEC/D3 brain endothelial cells.** The hCMEC/D3 brain endothelial cells expressing the lentiviral Gαi1 shRNA plus the lentiviral Gαi3 shRNA ("Gαi1/3-shRNA") or the scramble control shRNA ("scr-shRNA") were established; Cells were treated with RSOP3 (50 ng/mL) or the vehicle control ("Veh") and were further cultured for the designated time periods, and expression of listed proteins was shown (**A** and **B**); Cell migration ("Transwell" assays, **C**) and proliferation (by testing EdU-positive nuclei ratio, **D**) were tested by the listed assays. Data were presented as mean ± standard deviation (SD, n = 5). "No-shRNA" stands for the parental control cells without shRNA infection. * ***P*** < 0.05 versus "scr-shRNA" cells. # ***P*** < 0.05 versus "Veh" treatment. The experiments were repeated five times with similar results obtained. Scale bar = 100 μm.

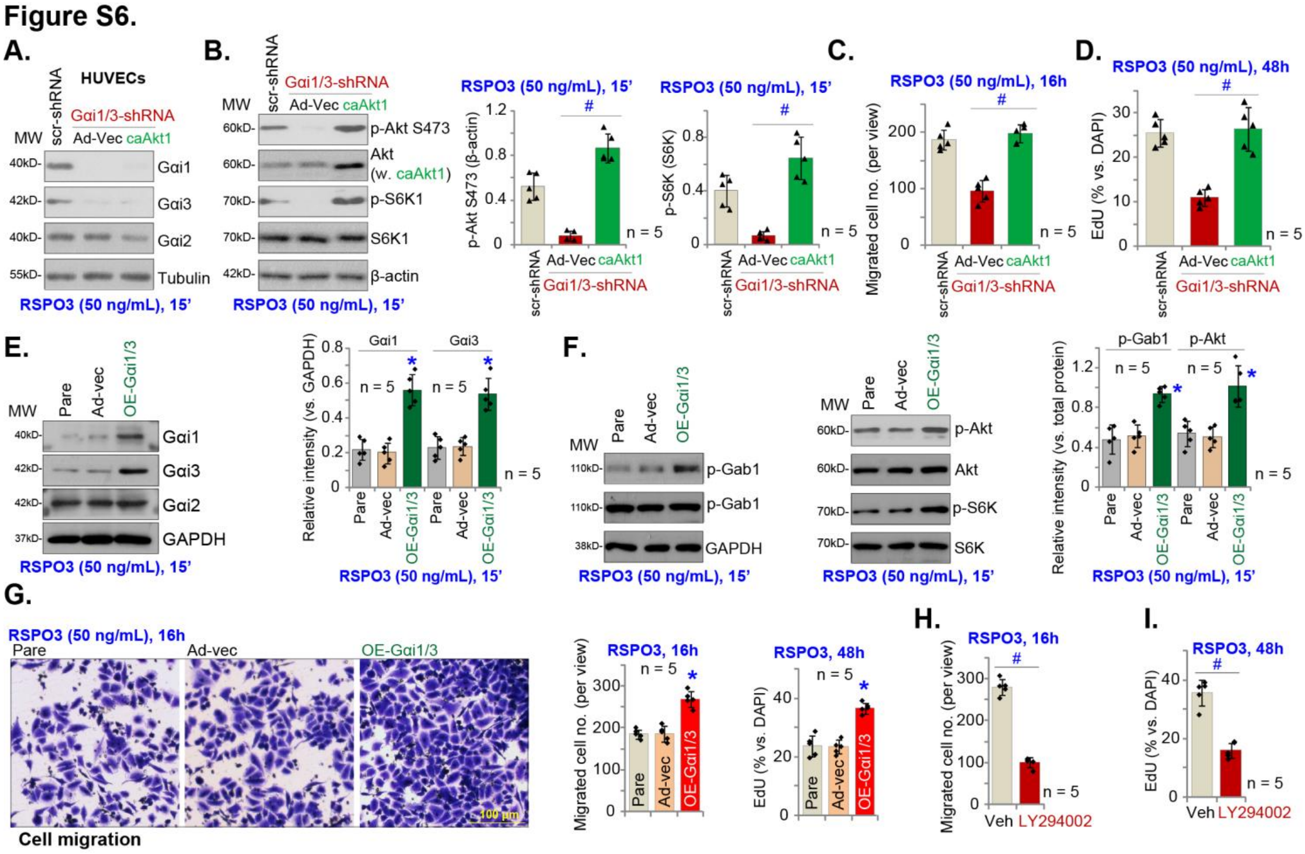

Figure S6. Gαi1/3 mediation of RSPO3-induced pro-angiogenic functions is due to transducing Akt-mTOR signaling. Stable HUVECs expressing the lentiviral Gαi1 shRNA plus the lentiviral Gαi3 shRNA ("Gαi1/3-shRNA") were further infected with the adenovirus encoding the constitutively-active Akt1 (ca-Akt1, S473D) or the empty vector ("Ad-Vec"), and stable cells were established after selection; Control HUVECs were with scramble control shRNA ("scr-shRNA"). HUVECs were treated with RSOP3 (50 ng/mL) or the vehicle control ("Veh") and further cultured for the designated time periods, and expression of listed proteins was shown, and protein phosphorylation was quantified (**A** and **B**); Cell migration ("Transwell" assays, **C**) and proliferation (by testing EdU-positive nuclei ratio, **D**) were tested. The stable HUVECs expressing the adenoviral Gαi1 expressing construct plus the adenoviral Gαi3 expressing construct (OE-Gαi1/3) or the empty vector ("Ad-Vec") were established. Cells were then treated with RSOP3 (50 ng/mL) and were further cultured for the designated time periods, expression of listed proteins was shown and results were quantified (**E** and **F**); Cell migration and proliferation (by testing EdU-positive nuclei ratio) were tested by the listed assays (**G**). OE-Gαi1/3 HUVECs were pretreated for 1h with LY294002 (5 μM) or the DMSO control ("Veh"), followed by RSOP3 (50 ng/mL) treatment and further cultured for the designated time periods; Cellular functions were tested and results were quantified (**H** and **I**). Data were presented as mean ± standard deviation (SD, n=5). "Pare" stands for the parental control cells. #

$P < 0.05$ (**B**-**D**, **H** and **I**). * $P < 0.05$ versus "Pare" cells (**E**-**G**). The experiments were repeated five times with similar results obtained. Scale bar = 100 μm.

Figure S7.

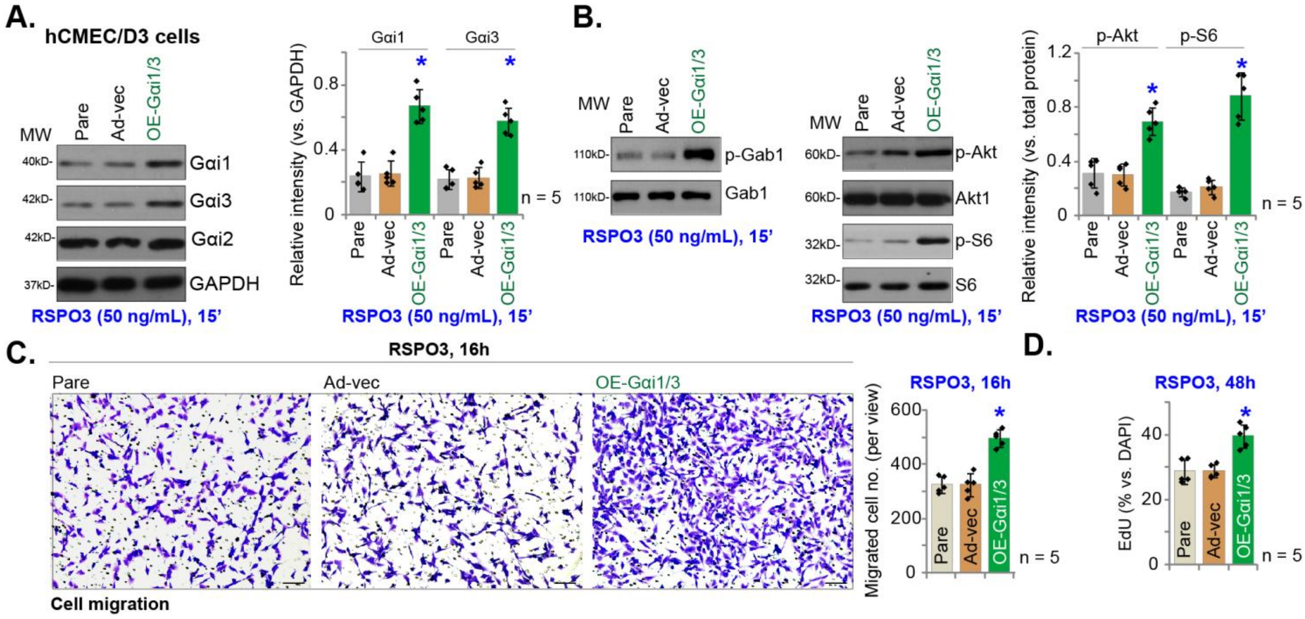

Figure S7. Gαi1/3 overexpression enhances RSPO3-induced pro-angiogenic functions in hCMEC/D3 brain endothelial cells. The hCMEC/D3 brain endothelial cells expressing the adenoviral Gαi1 expressing construct plus the adenoviral Gαi3 expressing construct (OE-Gαi1/3) or the empty vector ("Ad-Vec") were established. Cells were then treated with RSOP3 (50 ng/mL) and cultured for the designated time periods, and expression of listed proteins was shown (**A** and **B**); Cell migration ("Transwell" assays, **C**) and proliferation (by testing EdU-positive nuclei ratio, **D**) were tested. The data were presented as mean ± standard deviation (SD, n = 5). * $P < 0.05$ versus "Ad-Vec" cells. The experiments were repeated five times with similar results obtained. Scale bar = 100 μm.

References

Bai, J.Y., Li, Y., Xue, G.H., Li, K.R., Zheng, Y.F., Zhang, Z.Q., Jiang, Q., Liu, Y.Y., Zhou, X.Z., and Cao, C. (2021). Requirement of Galphai1 and Galphai3 in interleukin-4-induced signaling, macrophage M2 polarization and allergic asthma response. Theranostics 11, 4894-4909.

Bian, Z.J., Shan, H.J., Zhu, Y.R., Shi, C., Chen, M.B., Huang, Y.M., Wang, X.D., Zhou, X.Z., and Cao, C. (2022). Identification of Galphai3 as a promising target for osteosarcoma treatment. Int J Biol Sci 18, 1508-1520.

Cao, C., Huang, X., Han, Y., Wan, Y., Birnbaumer, L., Feng, G.S., Marshall, J., Jiang, M., and Chu, W.M. (2009). Galpha(i1) and Galpha(i3) are required for epidermal growth factor-mediated activation of the Akt-mTORC1 pathway. Sci Signal 2, ra17.

Li, X., Wang, D., Chen, Z., Lu, E., Wang, Z., Duan, J., Tian, W., Wang, Y., You, L., Zou, Y.*, et al.* (2015). Galphai1 and Galphai3 regulate macrophage polarization by forming a complex containing CD14 and Gab1. Proc Natl Acad Sci U S A 112, 4731-4736.

Liu, Y.Y., Chen, M.B., Cheng, L., Zhang, Z.Q., Yu, Z.Q., Jiang, Q., Chen, G., and Cao, C. (2018). microRNA-200a downregulation in human glioma leads to Galphai1 over-expression, Akt activation, and cell proliferation. Oncogene 37, 2890-2902.

Lv, Y., Wang, Y., Song, Y., Wang, S.S., Cheng, K.W., Zhang, Z.Q., Yao, J., Zhou, L.N., Ling, Z.Y., and Cao, C. (2021). LncRNA PINK1-AS promotes G alpha i1-driven gastric cancer tumorigenesis by sponging microRNA-200a. Oncogene 40, 3826-3844.

Marshall, J., Zhou, X.Z., Chen, G., Yang, S.Q., Li, Y., Wang, Y., Zhang, Z.Q., Jiang, Q., Birnbaumer, L., and Cao, C. (2018). Antidepression action of BDNF requires and is mimicked by Galphai1/3 expression in the hippocampus. Proc Natl Acad Sci U S A 115, E3549-E3558.

Sun, J., Huang, W., Yang, S.F., Zhang, X.P., Yu, Q., Zhang, Z.Q., Yao, J., Li, K.R., Jiang, Q., and Cao, C. (2018). Galphai1 and Galphai3mediate VEGF-induced VEGFR2 endocytosis, signaling and angiogenesis. Theranostics 8, 4695-4709.

Wang, Y., Liu, Y.Y., Chen, M.B., Cheng, K.W., Qi, L.N., Zhang, Z.Q., Peng, Y., Li, K.R., Liu, F., Chen, G.*, et al.* (2021). Neuronal-driven glioma growth requires Galphai1 and Galphai3. Theranostics 11, 8535-8549.

Yao, J., Wu, X.Y., Yu, Q., Yang, S.F., Yuan, J., Zhang, Z.Q., Xue, J.S., Jiang, Q., Chen, M.B., Xue, G.H.*, et al.* (2022). The requirement of phosphoenolpyruvate carboxykinase 1 for angiogenesis in vitro and in vivo. Sci Adv 8, eabn6928.

Zhang, X.P., Li, K.R., Yu, Q., Yao, M.D., Ge, H.M., Li, X.M., Jiang, Q., Yao, J., and Cao, C. (2018). Ginsenoside Rh2 inhibits vascular endothelial growth factor-induced corneal neovascularization. FASEB J 32, 3782-3791.

Zhang, Y.M., Zhang, Z.Q., Liu, Y.Y., Zhou, X., Shi, X.H., Jiang, Q., Fan, D.L., and Cao, C. (2015). Requirement of Galphai1/3-Gab1 signaling complex for keratinocyte growth factor-induced PI3K-AKT-mTORC1 activation. J Invest Dermatol 135, 181-191.
